# With-No-Lysine Kinase 1 (WNK1) Augments TRPV4 Function in the Aldosterone-Sensitive Distal Nephron

**DOI:** 10.3390/cells10061482

**Published:** 2021-06-12

**Authors:** Viktor N. Tomilin, Kyrylo Pyrshev, Naghmeh Hassanzadeh Khayyat, Oleg Zaika, Oleh Pochynyuk

**Affiliations:** Department of Integrative Biology and Pharmacology, The University of Texas Health Science Center at Houston, 6431 Fannin, Houston, TX 77030, USA; Viktor.Tomilin@uth.tmc.edu (V.N.T.); Kyrylo.A.Pyrshev@uth.tmc.edu (K.P.); Naghmeh.HassanzadehKhayyat@uth.tmc.edu (N.H.K.); Oleg.L.Zaika@uth.tmc.edu (O.Z.)

**Keywords:** renal tubule, ion transport, K^+^ secretion, mechanosensitivity

## Abstract

Kidneys play a central role in regulation of potassium homeostasis and maintenance of plasma K^+^ levels within a narrow physiological range. With-no-lysine (WNK) kinases, specifically WNK1 and WNK4, have been recognized to regulate K^+^ balance, in part, by orchestrating maxi K^+^ channel (BK)-dependent K^+^ secretion in the aldosterone-sensitive distal nephron (ASDN), which includes the connecting tubule and collecting duct. We recently demonstrated that the Ca^2+^-permeable TRPV4 channel is essential for BK activation in the ASDN. Furthermore, high K^+^ diet increases TRPV4 activity and expression largely in an aldosterone-dependent manner. In the current study, we aimed to test whether WNK kinases contribute to regulation of TRPV4 activity and its stimulation by aldosterone. Systemic inhibition of WNK with WNK463 (1 mg/kgBW for 3 days) markedly decreased TRPV4-dependent Ca^2+^ influx in freshly isolated split-opened collecting ducts. Aldosterone greatly increased TRPV4 activity and expression in cultured mpkCCD_c14_ cells and this effect was abolished in the presence of WNK463. Selective inhibition of WNK1 with WNK-in-11 (400 nM, 24 h) recapitulated the effects of WNK463 on TRPV4-dependent Ca^2+^ influx. Interestingly, WNK-in-11 did not interfere with up-regulation of TRPV4 expression by aldosterone, but prevented translocation of the channel to the apical plasma membrane. Furthermore, co-expression of TRPV4 and WNK1 into Chinese hamster ovary (CHO) cells increased the macroscopic TRPV4-dependent cation currents. In contrast, over-expression of TRPV4 with a dominant negative WNK1 variant (K233M) decreased the whole-cell currents, suggesting both stimulatory and permissive roles of WNK1 in regulation of TRPV4 activity. Overall, we show that WNK1 is essential for setting functional TRPV4 expression in the ASDN at the baseline and in response to aldosterone. We propose that this new mechanism contributes to regulation of K^+^ secretion and, by extension, urinary K^+^ levels to maintain systemic potassium homeostasis.

## 1. Introduction

Transient receptor potential vanilloid type 4 (TRPV4) is a mechanosensitive Ca^2+^-permeable channel, which can be activated by a broad spectrum of physical and chemical stimuli, such as hypotonicity, flow-induced shear stress, arachidonic acid metabolites, 4α-phorbol ester derivatives, and moderately warm temperatures [1,2,3]. TRPV4 is ubiquitously expressed in many tissues and organs, although the highest expression levels have been found in the kidney [4]. Here, TRPV4 is predominantly localized to the connecting tubule and the collecting duct [5], which along with the distal convoluted tubule form the aldosterone-sensitive distal nephron (ASDN). Abundant cumulative evidence shows that TRPV4 serves as a mechanical sensor of tubular flow in the ASDN [5,6,7,8,9]. TRPV4 dysfunction compromises [Ca^2+^]_i_ homeostasis and abolishes flow-induced elevations of [Ca^2+^]_i_ in native freshly isolated split-opened ASDN of rodents and in primary cultures of human distal nephron cells [5,10,11].

It has been generally recognized that ASDN is critical for maintaining systemic Na^+^ and K^+^ homeostasis in response to variations in electrolyte intake [12,13,14]. Dietary Na^+^ restriction and K^+^ load similarly elevate circulating aldosterone levels but allow renal sodium conservation and kaliuresis, respectively, without major disturbances in the handling of the counterpart cation [15]. This is thought to be due to the presence of the recently discovered “potassium switch” mechanism orchestrated by the with-no-lysine kinases (WNKs) being downstream effectors of aldosterone and Angiotensin II [16]. During hypovolemia, WNK1 and WNK4 are shown to stimulate sodium chloride cotransporter (NCC) in the distal convoluted tubule and the epithelial Na^+^ channel (ENaC) in the collecting duct to augment Na^+^ reabsorption, while both kinases inhibit activity of the apical ROMK (K_ir_1.1) channel to limit K^+^ secretion in the ASDN [17]. On the other side, an increase in extracellular K^+^ levels after dietary potassium load depolarizes basolateral membrane of the distal convoluted tubule cells, causing Cl^−^-dependent inhibition of WNKs and decreased NCC activity and increased flow/fluid delivery to the collecting duct [18]. It was further shown that WNK1 stimulates activity and expression of the Ca^2+^-activated BK channel [19,20]. BK activity underlies the phenomenon of the flow-induced K^+^ secretion in the ASDN, thus favoring kaliuresis [21,22]. The critical role of WNKs in regulation of renal Na^+^ and K^+^ handling has been culminated by the seminal discovery that patients with pseudohypoaldosteronism type II (PHAII), also known as Gordon’s syndrome exhibit a rare combination of hypertension and hyperkalemia due to gain-of-function mutations in WNK1 and WNK4, which causes defective degradation of these kinases [23,24,25]. Furthermore, recently developed orally bioavailable inhibitor of WNKs, namely WNK463, was capable of decreasing blood pressure and augmenting urinary production in rodent hypertensive models [26].

Flow-induced K^+^ secretion via BK channel requires mechanosensitive elevations of [Ca^2+^]_i_ implying an essential role of TRPV4 in this process. Indeed, genetic deletion of TRPV4 abolishes flow-dependent net K^+^ transport in the perfused collecting ducts [6] and decreases single channel BK activity [9]. Furthermore, high dietary K^+^ intake markedly increases TRPV4 expression and activity depending on mineralocorticoid receptors, which strongly implies the role of aldosterone [9]. Importantly, TRPV4-/- mice develop renal K^+^ retention and hyperkalemia when fed with high K^+^ diet, suggesting its critical role in maintaining systemic K^+^ balance [9]. However, virtually nothing is known about intracellular signaling mechanisms controlling TRPV4 activity in the ASDN.

Considering a strong premise that TRPV4 channel is crucial for regulation of K^+^ balance, we tested the idea that aldosterone controls TRPV4 activity and expression in a WNK-dependent manner. In this study, we show that inhibition of WNKs and particularly WNK1 greatly reduces TRPV4 activity in both freshly isolated and cultured murine collecting duct cells. The kinase domain WNK1 is essential for stimulation of TRPV4 activity and expression by aldosterone.

## 2. Materials and Methods

### 2.1. Reagents and Animals

All chemicals and materials were from Sigma (St. Louis, MO, USA), VWR (Radnor, PA, USA), Fisher (Waltham, MA, USA), and Tocris (Ellisville, MO, USA) unless noted otherwise and were at least of reagent grade. Animal use and welfare adhered to the NIH Guide for the Care and Use of Laboratory Animals following protocols reviewed and approved by the Animal Care and Use Committees of the University of Texas Health Science Center at Houston (protocol # AWC 18-0093). For experiments, 6–10 weeks old C57BL/6 (WT) and TRPV4 -/- (with C57BL/6 background) mice were used. Generation and usage of TRPV4 -/- mice was described previously [9,27]. As necessary for experimental design, mice were given drinking water containing WNK463 (1 mg/kgBW) for 3 days, as described previously [26].

### 2.2. Isolation and Split-Opening of Collecting Ducts

The procedure for isolation of the collecting ducts from mouse kidneys suitable for fluorescent [Ca^2+^]_i_ measurements closely followed the protocols previously published by our group [28,29]. Kidneys were cut into thin slices (<1 mm) with slices placed into an ice-cold bath solution contained (in mM/L): 150 NaCl, 5 KCl, 1 CaCl_2_, 2 MgCl_2_, 5 glucose and 10 HEPES (pH 7.35). Collecting ducts were visually identified by their morphological features (pale color; coarse surface and, in some cases, bifurcations) and were mechanically isolated from kidney slices by micro-dissection using watchmaker forceps under a stereomicroscope. Isolated collecting ducts were attached to 5 mm × 5 mm cover glasses coated with poly L-lysine. A cover-glass containing a collecting duct was placed in a perfusion chamber mounted on an inverted Nikon Eclipse Ti microscope and perfused with bath solution at room temperature. Collecting ducts were further split-opened with two sharp micropipettes, controlled with different micromanipulators, to gain access to the apical membrane. The collecting ducts were used within 2 h of isolation.

### 2.3. Cell Culture

Immortalized mouse cortical collecting duct (mpkCCD_c14_) principal cells (originally described in [30]) were grown to confluency in DMEM/F12 medium (Cat. No. 10-092-CM, Cellgro, Tewksbury, MA, USA), supplemented with 3% FBS, 100 I.U./mL penicillin and 100 μg/mL streptomycin and 50 nM dexamethasone. Twenty four hours prior to experimentation, the medium was replaced with minimal medium that contained only DMEM, Ham’s F-12 and antibiotics. Vehicle, aldosterone (1 µM), cortisone (1 µM), WNK463 (100 nM), and WNK-in-11 (400 nM) were added to the media for 24 h, as was necessary for experimental setup.

### 2.4. [Ca^2+^]_i_ Imaging

Intracellular calcium levels were measured in freshly isolated split-opened collecting ducts or confluent mpkCCD_c14_ monolayers using Fura-2 fluorescence ratiometric imaging as described previously [10,29,31]. Briefly, the cells were loaded with Fura-2 by incubation with 2 μM Fura-2/AM in a bath solution for 40 min at room temperature. Subsequently, tissue samples were washed and incubated for additional 10–15 min prior to experimentation. Cover glasses containing a split-opened collecting duct or a mpkCCD_c14_ monolayer were placed in an open-top imaging study chamber (RC-26GLP; Warner Instruments, Hamden, CT, USA) with a bottom coverslip viewing window and the chamber attached to the microscope stage of a Nikon Ti-S Wide-Field Fluorescence Imaging System (Nikon Instruments, Melville, NY, USA) integrated with Lambda XL light source (Sutter Instrument, Novato, CA, USA) and QIClick 1.4 megapixel monochrome CCD camera (QImaging, Surrey, BC, Canada) via NIS Elements 4.3 Imaging Software (Nikon Instruments, Melville, NY, USA). Cells were imaged with a 40X Nikon Super Fluor objective and regions of interest (ROIs) were drawn for individual cells. The Fura 2 fluorescence intensity ratio was determined by excitation at 340 nm and 380 nm and calculating the ratio of the emission intensities at 511 nm in the usual manner every 5 s. The changes in the ratio are reported as an index of changes in intracellular calcium using Grynkiewicz formula, as well-documented previously [32]. F_min_ and F_max_ were determined in Fura-2 loaded split-opened collecting ducts treated with ionomycin (5 µM), when bath solution contained 0 and 2 mM Ca^2+^, respectively. No significant Fura-2 bleaching and leakage were detected during the timeline of experiments. At least three different collecting ducts (30–40 cells in each) from at least different three mice or five monolayers (approximately 100 cells in each) of mpkCCD_c14_ cells were used for each experimental condition.

### 2.5. Isolation of TRPV4 Membrane Fraction

To assess TRPV4 abundance at the plasma membrane, giant plasma membrane vesicles (GPMV) were isolated from confluent mpkCCD_c14_ monolayers according to previously described protocol [33]. Briefly, cells were extensively washed with PBS and further treated with 25 mM PFA (paraformaldehyde, AzerScientific, Morgantown, PA, USA) with 2 mM DTT (1,4-Dithiothreitol, SigmaAldrich, Burlington, VT, USA) at +37 °C for 1 h to stimulate GPMV formation. Production of the vesicles was controlled with a microscope at 20× magnification. GPMV were observed as the free-floating spherical objects (with the average size of 5 µM). The vesicles were collected and further centrifuged at 100 RCF for 10 min to eliminate the cellular debris. The supernatant was sedimented at 20,000 RCF for 1 h at +4 °C with Sorvall RC5C plus ultra-centrifuge. The pellet was collected and used for Western blot analysis.

### 2.6. Western Blotting

Immediately after dissection kidneys were placed on ice, decapsulated and homogenized in three volumes of ice-cold M-PER Mammalian Protein Extraction Reagent (Thermo scientific, Houston, TX, USA) supplemented with Complete Mini protease and PhosSTOP phosphatase inhibitor cocktails (Roche Diagnostics, Indianapolis, IN, USA). mpkCCD_c14_ cell culture lysates were obtained by scraping cells off 100 mm Petri dishes and homogenizing in 400 µL of Mammalian Protein Extraction Reagent with protease and phosphatase inhibitors. Protein concentration was determined with IMPLEN NanoPhotometer N60 with a standard UV-based protocol. The samples were additionally diluted when necessary with Mammalian Protein Extraction Reagent, denatured and reduced in Laemmli buffer supplemented with 5% β-ME at +75 °C for 10 min to reach the final protein concentration of approximately 2 mg/mL. The samples (40 μg/lane for kidney and cellular samples, 20 μg/lane for GPMV samples) were separated on 9% polyacrylamide resolving gels at 175 V for 150 min and transferred to nitrocellulose membrane for 110 min at 100 V. Equal protein load was further verified by Ponceau red staining using standard procedures. Subsequently the nitrocellulose membrane was pre-blocked with 5% milk in TBS-Tween for 1 h at +4C and further incubated with anti-TRPV4 (1:1000, Alomone labs, Jerusalem, Israel) antibodies overnight at +4 °C. Upon washout (3 times for 10 min in TBS-Tween), the membrane was incubated with peroxidase-conjugated goat anti-rabbit (1:10000, Jackson ImmunoResearch Laboratories, USA) secondary antibodies for 1 h at +4 °C. The signal was produced by SuperSignal West Dura Extended duration substrate (Thermo scientific, USA) and recorded by Bio-Rad ChemiDoc MP Imaging system. Blots were quantified using ImageJ 1.50e software (NIH, Bethesda, MD, USA). The intensities of the studied protein bands were normalized to the total signal of the respective line in Ponceau red staining.

### 2.7. Over-Expression of TRPV4 in Chinese Hamster Ovary (CHO) Cells

CHO cells were obtained from the American Type Culture Collection. These cells were maintained with standard culture conditions (Dulbecco’s modified Eagle’s medium + 10% fetal bovine serum, 37 °C, 5% CO_2_). Overexpression of mouse TRPV4 and WNK1 into CHO cells was performed by transfecting the appropriate expression plasmids using the Polyfect reagent (Qiagen, Valencia, CA, USA) following manufacturer’s protocols, as described previously [34]. Transfected cells were identified by GFP fluorescence after co-expression with green fluorescent protein (GFP) cDNA. Electrophysiological experiments were performed 48–72 h after transfection. For studies, 0.8 µg/9.6 cm^2^ of TRPV4/WNK1/WNK1 (K233M) and 0.5 µg/9.6 cm^2^ of all other plasmids cDNA were used. The cDNA encoding WNK1 and DN-WNK1 (K233M) were described previously [34,35]. Whole-cell capacitance was routinely compensated and was approximately 9 pF for CHO cells. Series resistances, on average 2–5 MegaOhm, were also compensated. Currents were evoked with 1 sec ramp protocol from −80 to +60 mV. Bath and pipette solutions were (in mM/L): 150 NaCl, 5 KCl, 1 CaCl_2_, 2 MgCl_2_, 5 glucose and 10 HEPES (pH 7.35); and 150 KCl, 5 NaCl, 2 MgCl_2_, 5 EGTA, 10 HEPES, 2 ATP, 0.1 GTP (pH 7.35) respectively. Upon recording baseline currents for 2 min, TRPV4 agonist GSK1016790A (40 nM) was applied for 5 min followed by TRPV4 antagonist GSK2798745 (40 nM) for 3 min.

### 2.8. Data Analysis and Presentation

All summarized data are reported as mean ± SEM. Statistical comparisons were made using one-way ANOVA with post hoc Tukey test or one-way repeated measures ANOVA with post hoc Bonferroni test (for paired experiments within the same group). *p* value less than 0.05 was considered significant. The principal scheme was prepared using the templates from Servier Medical Art web-page (licensed under a Creative Commons Attribution 3.0 Unported License).

## 3. Results

### 3.1. WNK Blockade Decreases TRPV4 Activity and Expression in the ASDN

**Figure 1:**
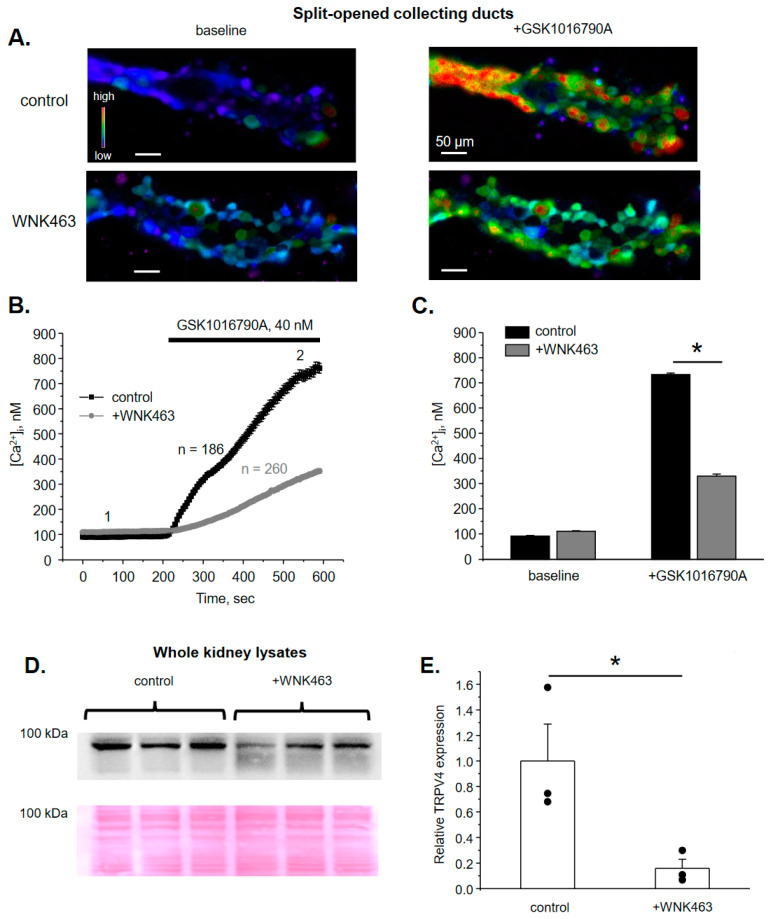
WNK inhibition decreases TRPV4 activity and expression in ASDN. (**A**) Representative pseudo-color images of [Ca^2+^]_i_ changes (blue—low and red—high) in isolated split-opened collecting ducts loaded with Ca^2+^-sensitive dye Fura2 at the baseline (left) and following 5 min application of TRPV4 agonist, GSK1016790A (right) from control (top) and WNK463 treated (bottom) mice. (**B**) The averaged time-courses of [Ca^2+^]_i_ changes upon application of 40 nM GSK1016790A (shown with the bar on top) in individual collecting duct cells within split-opened area from control and WNK463 treated mice. The time-points 1 (baseline) and 2 (GSK1016790A) represent conditions shown in panel (A). (**C**) Summary graph comparing [Ca^2+^]_i_ values in individual collecting duct cells from control and WNK463 treated mice at the baseline and after GSK1016790A application. *—significant difference (*p* < 0.05) between groups indicated with a line. (**D**) Actual Western blot probed with anti-TRPV4 antibodies from whole kidney lysates from mice kept on standard conditions (control) or treated with WNK463. The Ponceau red staining of the same nitrocellulose membrane demonstrating equal protein loading is shown on the bottom panel. (**E**) Summary graph comparing TRPV4 expression levels in the conditions from panel (**D**). The intensity values were normalized to the total signal of the respective lines in Ponceau red staining. *—significant differences (*p* < 0.05).

Ca^2+^-permeable TRPV4 channel is abundantly expressed in the ASDN, where it is critical for flow sensitivity as well as for regulation of K^+^ secretion via BK channel [5,6,9]. WNKs, particularly WNK1 and WNK4, are recognized to orchestrate a variety of Na^+^ and K^+^ transporting systems in the ASDN and the early distal convoluted tubule (DCT) [16,17]. Thus, we first tested whether TRPV4 activity in the ASDN is controlled by WNKs. As shown in the representative image of a split-opened collecting duct loaded with Ca^2+^-sensitive dye Fura2 (Figure 1A) and the respective time course of [Ca^2+^]_i_ changes (Figure 1B), TRPV4 agonist, GSK1016790A (40 nM) induces a robust elevation of [Ca^2+^]_i_ from 91 ± 2 nM to 733 ± 6 nM at 5 min of application. Of note, GSK1016790A has no effect on [Ca^2+^]_i_ levels in split-opened collecting ducts isolated from TRPV4-/- mice (Supplementary Figure S1) providing a direct proof that GSK1016790A increases [Ca^2+^]_i_ exclusively in a TRPV4-dependent manner in collecting duct cells. Systemic blockade of WNK signaling cascade with the orally bioavailable pan-WNK-kinase inhibitor, WNK463 (1 mg/kgBW) for 3 days dramatically attenuates GSK1016790A-induced [Ca^2+^]_i_ elevations from 110 ± 3 nM to 329 ± 7 nM (Figure 1B,C). This was also associated with a marked reduction in renal TRPV4 levels, as is shown on the Western blot (Figure 1D) and the respective summary graph (Figure 1E). TRPV4-reporting signal was absent in kidney lysates from TRPV4-/- mice thus verifying specificity of the used antibodies (Figure S2). Altogether, these data suggest a critical role of WNK signaling in setting TRPV4 activity in ASDN cells.

### 3.2. WNK1 Is Critical for Stimulation of TRPV4 by Aldosterone in Cultured mpkCCD_c14_ Cells

**Figure 2:**
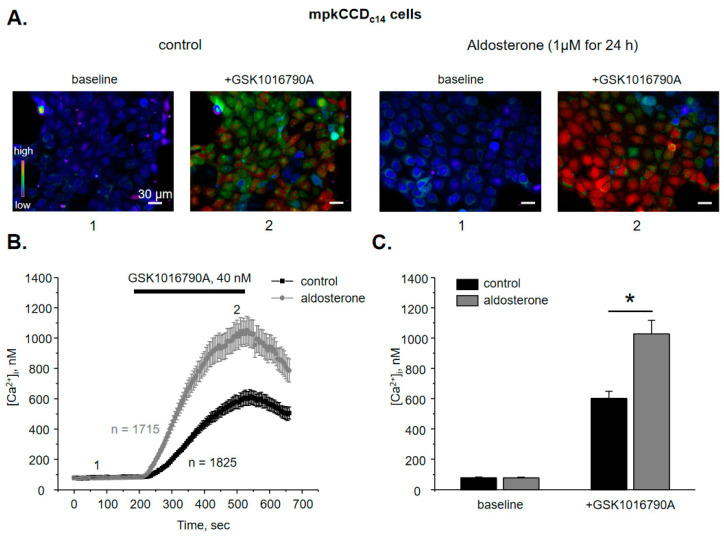
Aldosterone augments TRPV4-dependent Ca^2+^ influx in mpkCCD_c14_ cells. (**A**) Representative pseudo-color images of [Ca^2+^]_i_ changes (blue—low and red—high) in confluent monolayers of mpkCCD_c14_ cells at the baseline (1) and following 5 min application of TRPV4 agonist, GSK1016790A (2) maintained on standard (control) conditions and following 24 h treatment with aldosterone. (**B**) The averaged time-courses of [Ca^2+^]_i_ changes in individual mpkCCD_c14_ cells in the control and after incubation with aldosterone upon application of 40 nM GSK1016790A (shown with the bar on top). The time-points 1 (baseline) and 2 (GSK1016790A) represent conditions shown in panel (A). (**C**) Summary graph comparing [Ca^2+^]_i_ values in individual mpkCCD_c14_ cells in the control and following aldosterone treatment at the baseline and after GSK1016790A application. *—significant difference (*p* < 0.05) between groups is indicated with a line.

**Figure 3:**
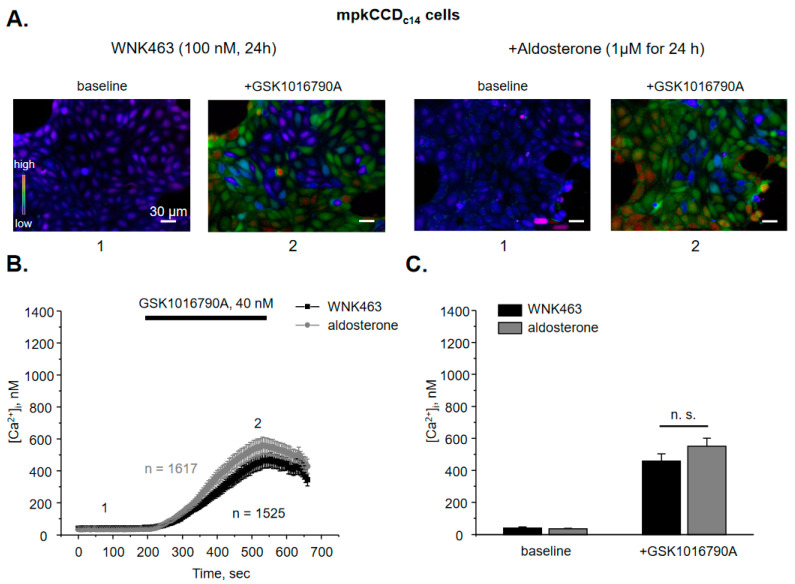
Inhibition of WNK signaling cascade precludes stimulation of TRPV4 activity by aldosterone in mpkCCD_c14_ cells. (**A**) Representative pseudo-color images of [Ca^2+^]_i_ changes (blue—low and red—high) at the baseline (1) and following 5 min application of TRPV4 agonist, GSK1016790A (2) in confluent monolayers of mpkCCD_c14_ cells treated with pan-specific WNK inhibitor WNK463 (100 nM) in the absence and presence of concomitant administration of aldosterone. (**B**) The averaged time-courses of [Ca^2+^]_i_ changes in individual WNK463 pretreated mpkCCD_c14_ cells in the absence and presence of aldosterone upon application of 40 nM GSK1016790A (shown with the bar on top). The time-points 1 (baseline) and 2 (GSK1016790A) represent conditions shown in panel (A). (**C**) Summary graph comparing [Ca^2+^]_i_ values in individual WNK463 pretreated mpkCCD_c14_ cells in the control and following aldosterone treatment at the baseline and after GSK1016790A application. N.s. represents non-significant difference (*p* > 0.05).

We have previously demonstrated that aldosterone increases TRPV4 activity and expression in the ASDN [9] but the signaling determinants of this regulation are not yet elucidated. Thus, we next tested whether aldosterone regulates TRPV4 function in a WNK-dependent manner. Since systemic inhibition of WNKs could exhibit both direct and indirect (extra-renal) actions on TRPV4, we next quantified the effects of aldosterone on TRPV4 in cultured mpkCCD_c14_ cells, a well-accepted model of the collecting duct principal cells [30]. Application of 40 nM GSK1016790A for 5 min induces a robust elevation of [Ca^2+^]_i_ in mpkCCD_c14_ cell monolayers (Figure 2A) from 78 ± 5 nM to 603 ± 40 nM (Figure 2B). Importantly, the time course and the magnitude of the response are comparable with the observed values in freshly isolated split-opened collecting ducts (Figure 1C and Figure 2C). Pretreatment of mpkCCD_c14_ cells with aldosterone (1 µM for 24 h) augments GSK1016790A-induced [Ca^2+^]_i_ responses nearly two-fold (Figure 2A,C). The basal and GSK1016790A-stimulated [Ca^2+^]_i_ values are 77 ± 5 nM and 1028 ± 89 nM, respectively. Interestingly, pretreatment with glucocorticoid, cortisone (1 µM for 24 h) similarly augments GSK1016790A [Ca^2+^]_i_ responses in mpkCCD_c14_ cells (Supplementary Figure S3), suggesting that the stimulatory actions are most likely mediated by the cytosolic steroid receptors. Inhibition of WNK signaling with pan-selective WNK inhibitor, WNK463 (100 nM for 24 h) significantly decreases both baseline [Ca^2+^]_i_ and GSK1016790A-induced responses to 41 ± 5 nM and 457 ± 45 nM, respectively (Figure 3A,B). Importantly, aldosterone fails to significantly augment TRPV4 activity when applied concurrently with WNK463 (Figure 3A–C). The mean values of [Ca^2+^]_i_ are 36 ± 3 nM at the baseline and 550 ± 51 nM after application of GSK1016790A. Overall, these results imply that regulation of TRPV4 activity by aldosterone in the ASDN cells depends on the functional status of WNK signaling cascade.

**Figure 4:**
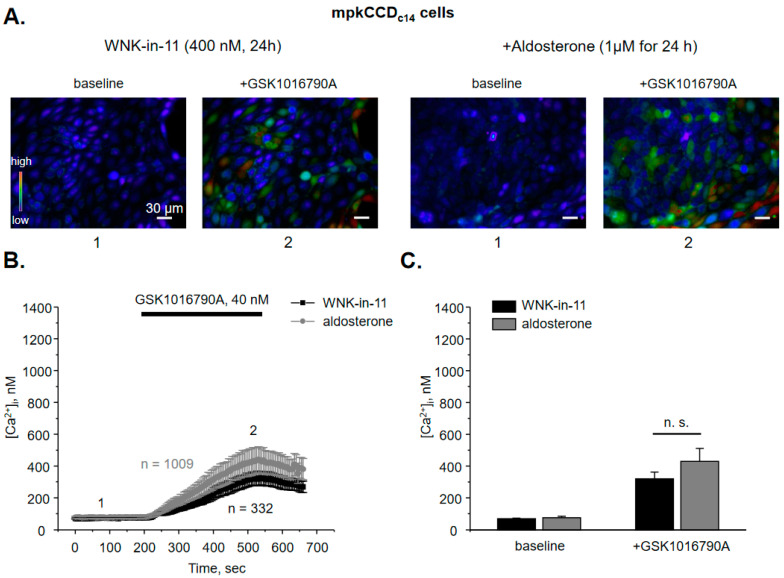
WNK1 is critical for stimulation of TRPV4 activity by aldosterone in mpkCCD_c14_ cells. (**A**) Representative pseudo-color images of [Ca^2+^]_i_ changes (blue—low and red—high) in confluent monolayers of mpkCCD_c14_ cells treated with the selective WNK1 inhibitor WNK-in-11 (400 nM) in the absence and presence of concomitant administration of aldosterone at the baseline (1) and following 5 min application of TRPV4 agonist, GSK1016790A (2). (**B**) The averaged time-courses of [Ca^2+^]_i_ changes upon application of 40 nM GSK1016790A (shown with the bar on top) in individual WNK-in-11 pretreated cells mpkCCD_c14_ cells in the absence and presence of aldosterone. The time-points 1 (baseline) and 2 (GSK1016790A) represent conditions shown in panel (A). (**C**) Summary graph comparing [Ca^2+^]_i_ values in individual WNK-in-11 pretreated mpkCCD_c14_ cells in the control and following aldosterone treatment at the baseline and after GSK1016790A application. N.s. represents non-significant difference (*p* > 0.05).

Both WNK1 and WNK4 are known to be expressed in the ASDN cells [16]. WNK1 was recently shown to increase activity and expression of BK channel [19,20], which is functionally coupled with TRPV4 [5,6,9]. Thus, we next tested whether WNK1 is also instrumental in controlling TRPV4 activity. As shown in the representative micrographs (Figure 4A) and the average time course of [Ca^2+^]_i_ changes (Figure 4B), pretreatment with WNK1 selective blocker, WNK-in-11 (400 nM, 24 h; [36]) significantly reduces GSK1016790A-mediated [Ca^2+^]_i_ responses with basal and TRPV4-dependent [Ca^2+^]_i_ values being at 69 ± 3 nM and 320 ± 42 nM, respectively. Furthermore, WNK-in-11 nearly precludes the stimulatory actions of aldosterone on GSK1016790A-dependent [Ca^2+^]_i_ responses: 75.5 ± 8 nM and 430 ± 80 nM (Figure 4C).

**Figure 5:**
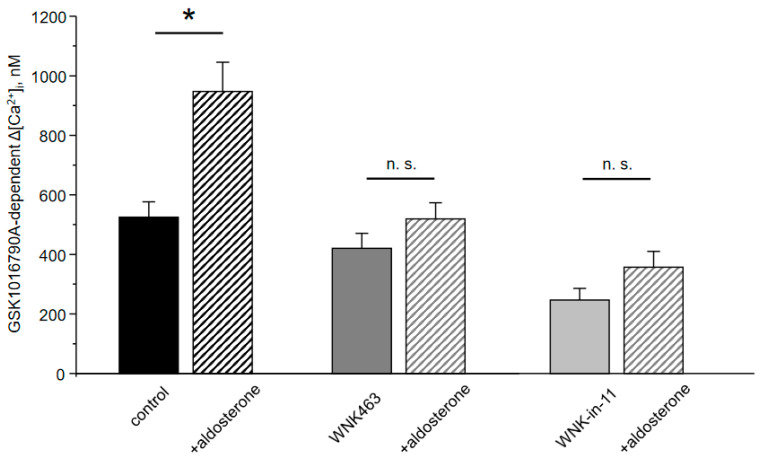
Inhibition of WNK1 abolishes regulation of TRPV4 activity by aldosterone in mpkCCD_c14_ cells. Summary graph comparing the magnitudes of GSK1016790A-mediated [Ca^2+^]_i_ elevations calculated as the difference in [Ca^2+^]_i_ values before and after application of the TRPV4 agonist in individual mpkCCD_c14_ cells kept on control conditions and treated with aldosterone, pan-specific WNK blocker WNK463, and selective WNK1 inhibitor WNK-in-11 as indicated. *—significant difference (*p* < 0.05) or n.s.—non-significant difference (*p* > 0.05) between groups is indicated with a line.

As summarized in Figure 5, WNK463 and WNK-in-11 tended to moderately decrease TRPV4-dependent Ca^2+^ influx at the baseline. Importantly, both antagonists preclude stimulation of TRPV4 activity by aldosterone. Based on the results in Figure 3, Figure 4 and Figure 5, we concluded that the selective inhibition of WNK1 recapitulates the effects of pan-specific inhibition of WNKs thus arguing for a central role of WNK1 in regulation of TRPV4 activity by aldosterone.

**Figure 6:**
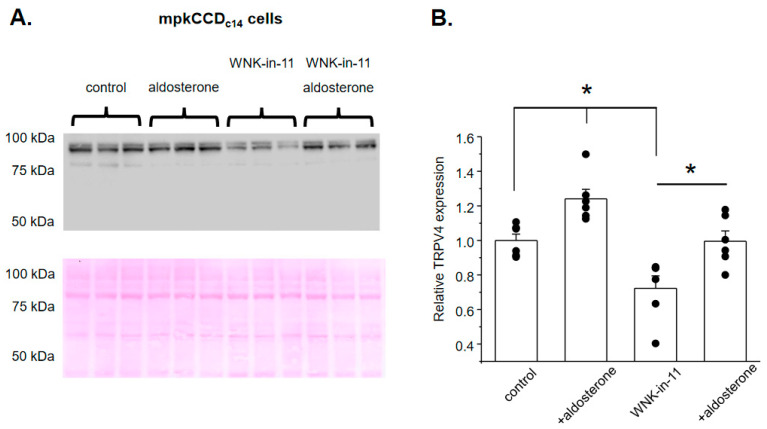
WNK1 blockade reduces TRPV4 expression but does not interfere with stimulation of TRPV4 abundance by aldosterone. (**A**) Representative Western blot from whole cell lysates probed with anti-TRPV4 antibodies in mpkCCD_c14_ cells kept on standard conditions (control), treated with aldosterone (1 µM for 24 h), WNK1 inhibitor WNK-in-11 (400 nM for 24 h), and aldosterone together with WNK-in-11, as is indicated on top. The Ponceau red staining of the same nitrocellulose membrane demonstrating equal protein loading is shown on the bottom panel. (**B**) Summary graph comparing TRPV4 expression levels in the conditions from panel (**A**). The intensity values were normalized to the total signal of the respective lines in Ponceau red staining. *—significant differences (*p* < 0.05) between groups are indicated with respective lines on top.

**Figure 7:**
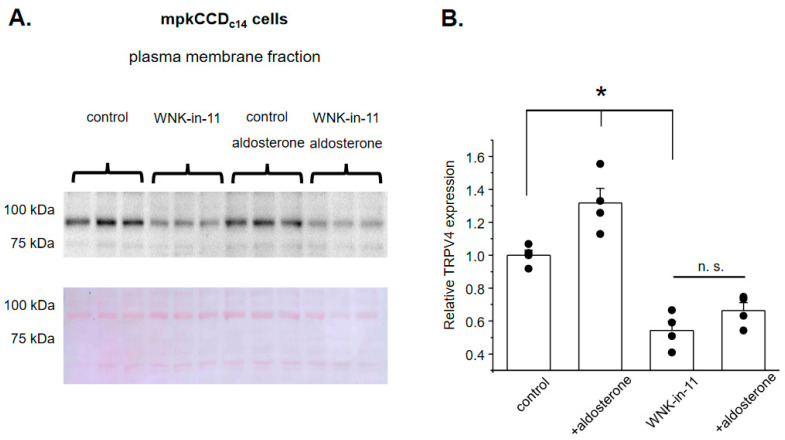
WNK1 governs translocation of TRPV4 to the plasma membrane in response to aldosterone. (**A**) Representative Western blot from the plasma membrane fraction probed with anti-TRPV4 antibodies in mpkCCD_c14_ cells kept on standard conditions (control), treated with aldosterone (1 µM for 24 h), WNK1 inhibitor WNK-in-11 (400 nM for 24 h), and aldosterone together with WNK-in-11, as is indicated on top. The membrane fraction was separated using the giant plasma membrane vesicles’ isolation technique, as described in the methods. The Ponceau red staining of the same nitrocellulose membrane demonstrating equal protein loading is shown on the bottom panel. (**B**) Summary graph comparing plasma membrane TRPV4 expression levels in the conditions from panel (A). The intensity values were normalized to the total signal of the respective lines in Ponceau red staining. *—significant differences (*p* < 0.05) or n.s—non-significant difference (*p* > 0.05) between groups are indicated with respective lines on top.

We next examined whether reduced GSK1016790A-dependent [Ca^2+^]_i_ responses upon WNK1 blockade are also associated with a decreased TRPV4 expression in cultured mpkCCD_c14_ cells. As shown on the representative Western blot from whole-cell lysates (Figure 6A), aldosterone (1 µM, 24 h) significantly increases TRPV4 levels, which is consistent with its stimulation of TRPV4-dependent Ca^2+^ influx (Figure 2). Interestingly, pretreatment with WNK-in-11 (400 nM, 24 h) significantly decreases basal TRPV4 levels but does not preclude up-regulation of TRPV4 expression by aldosterone (Figure 6A,B). To address these seemingly discrete effects of WNK1 inhibition on TRPV4 activity (Figure 4) and TRPV4 expression (Figure 6), we next monitored TRPV4 abundance on the apical plasma membrane using the giant plasma membrane vesicles (GPMV) approach [33] in confluent mpkCCD_c14_ monolayers in the control and after stimulation with aldosterone. As shown previously, GPMVs contain virtually pure plasma membrane fragments without any measurable intra-vesicular/intra-cellular components [37], thus being a reliable and convenient tool to track and quantify expression levels of various proteins on the plasma membrane. As shown on the representative Western blot and the summary graph (Figure 7A,B), the membrane TRPV4 levels are significantly increased in aldosterone-treated cells. Importantly, WNK-in-11 reduces TRPV4 abundance at the plasma membrane and abolishes the stimulatory effect of aldosterone (Figure 7A,B). Overall, our results demonstrate that WNK1 is critical for regulation of TRPV4 activity and translocation of the channel to the apical plasma membrane in response to aldosterone.

### 3.3. WNK1 Increases TRPV4 Activity in a Kinase-Dependent Manner

**Figure 8:**
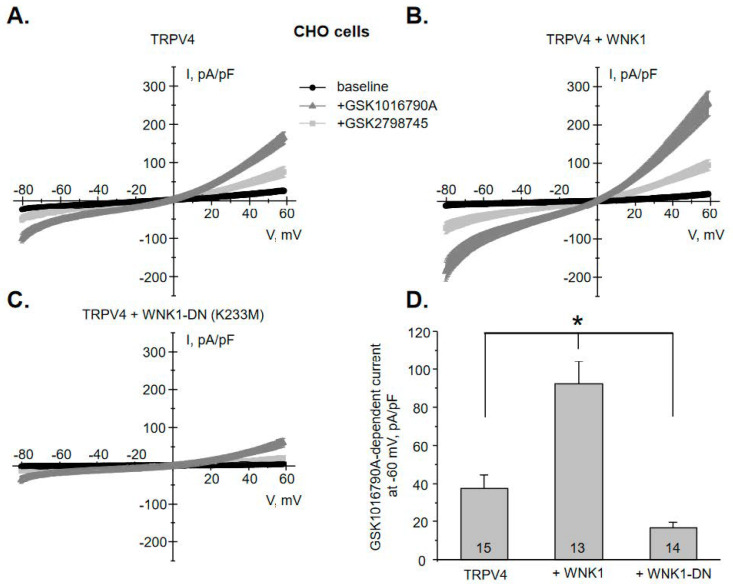
WNK1 regulates TRPV4 activity in a kinase-dependent manner. (**A**) Averaged macroscopic whole-cell current-voltage (I-V) relations in Chinese Hamster Ovary (CHO) cells transfected with TRPV4 at the baseline (black), following application of TRPV4 agonist GSK1016790A (40 nM) for 5 min (gray), and TRPV4 antagonist GSK2798745 (40 nM) for 3 min (light gray). Currents were evoked by a voltage ramp from −80 to +60 mV for 1 sec. The averaged whole-cell I-V relations upon transfection of TRPV4 with WNK1 (**B**) and kinase dead WNK1 dominant negative (DN) point mutation K233M (**C**). All other conditions are identical to those described in panel A. (**D**) The summary graph comparing the amplitudes of GSK1016790A-induced currents at −60 mV for CHO cells transfected with TRPV4, TRPV4 + WNK1, and TRPV4 + WNK1-DN. The number of individual experiments is shown for each condition. *—significant difference (*p* < 0.05) between groups is indicated with a line on top.

We next examined whether WNK1 is capable of directly increasing TRPV4 activity. For this, we quantified macroscopic GSK1016790A-induced currents in CHO cells upon over-expression of TRPV4. Figure 8A shows an average current-voltage (I-V) relation before (baseline) and after application of GSK1016790A (40 nM for 5 min). Non-transfected cells have no background currents [38] and do not respond to GSK1016790A application (Supplementary Figure S4A). GSK1016790A-induced macroscopic currents are moderately higher at positive voltages (outward rectification) with the reversal at 0 mV, which is a characteristic of a non-selective channel, such as TRPV4 [39]. GSK1016790A is a poorly reversible agonist. To ensure that these currents do not also include “leak” current due to putative decay of a high-GigaOhm contact between a recording pipette and a cell, TRPV4 antagonist, GSK2798745 (40 nM [40]) was subsequently applied. GSK2798745 decreases the current magnitude by approximately 70% after 3 min (Figure 8A) and by nearly 100% after 10 min (Supplementary Figure S4B). For the reason of efficiency, we considered that a patch has no notable “leak” if the majority of the current is blocked by GSK2798745 after 3 min. Co-expression of TRPV4 with WNK1 significantly augments GSK1016790A-dependent TRPV4 currents from 38 ± 7 pA/pF to 92 ± 12 pA/pF at −60 mV (Figure 8B). Importantly, expression of dominant negative (DN) kinase dead WNK1 mutant K233M [41,42] significantly decreases TRPV4 currents below the baseline values to 17 ± 3 pA/pF (Figure 8C). Figure 8D shows a summary graph comparing the magnitudes of GSK1016790A-induced current during the aforementioned conditions. Overall, our in vitro experiments demonstrate a strong positive correlation between TRPV4 activity and function of the kinase domain of WNK1.

## 4. Discussion

**Figure 9:**
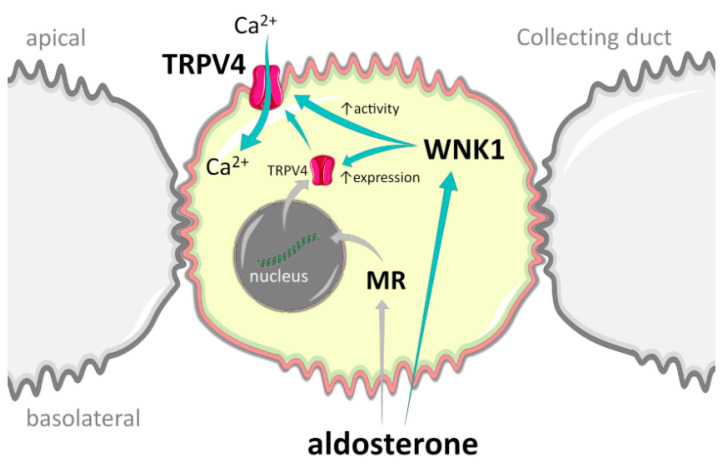
Principal scheme of TRPV4 regulation by WNK1 in the collecting duct cells. Green arrows represent stimulatory actions. MR—mineralocorticoid receptors.

In this manuscript, we identified a new mechanism of regulation of TRPV4 in the ASDN by aldosterone-MR-WNK1 signaling (Figure 9). We demonstrated that inhibition of WNK cascade and more specifically WNK1 abolishes stimulation of TRPV4 activity by aldosterone and precludes TRPV4 translocation to the plasma membrane. Furthermore, inactivation of WNK1 kinase activity reduces TRPV4 activity below control values, arguing for its essential role in regulation of both basal and aldosterone-induced TRPV4 function in the kidney.

We previously showed that TRPV4 expression and activity are up-regulated by elevated dietary K^+^ intake [9]. Furthermore, inhibition of mineralocorticoid receptors with spironolactone abolishes the stimulatory effects of K^+^-rich diet, thus implying a critical role of aldosterone [9]. Consistently, aldosterone treatment of the cultured mpkCCD_c14_ cells recapitulates the stimulatory actions of high K^+^ diet with respect to TRPV4 activity and expression (Figure 2 and Figure 6). While we cannot disqualify the direct effects of elevated K^+^ or increased flow, it appears that aldosterone serves as a major physiologically relevant TRPV4 effector in the kidney. It also seems that mineralocorticoid receptors mediate the stimulatory actions of aldosterone, since cortisone is also capable of stimulation TRPV4-dependent Ca^2+^ influx in mpkCCD_c14_ cells (Figure S3). Of interest, stimulation of glucocorticoid receptors can also lead to up-regulation of TRPV4 mRNA and its activity in extra-renal tissues [43,44], potentially indicating the common mode of channel activation by steroid hormones.

It was demonstrated that TRPV4 activity is necessary for the flow-induced K^+^ secretion via BK channel in the collecting duct [6,9]. Thus, it is reasonable to assume that stimulation of TRPV4 by aldosterone and WNK1 as reported here (Figure 4, Figure 6 and Figure 8) would favor potassium secretion. Consistently, it was shown that WNK1 increases expression of the pore-forming BK α subunit and BK channel activity by inhibiting ERK1/2-mediated lysosomal degradation of the channel in response to dietary K^+^ load [19,20]. On the other hand, WNK1 is known to inhibit apically localized potassium ROMK channel in the collecting duct [45,46]. Moreover, hyperkalemia during Gordon’s syndrome (aka PHAII, FHHt) is rather consistent with renal K^+^ retention when WNK1 is over-active [25]. This dissonant stimulation of BK and inhibition of ROMK channel by WNK1 in the collecting duct requires further elaboration. The “baseline” ROMK-mediated K^+^ secretion almost exclusively depends on Na^+^ delivery to the collecting duct, followed by Na^+^ entry via ENaC [14]. Thus, stimulation of NCC in the distal convoluted tubule and ENaC in the collecting duct allows efficient Na^+^ conservation during hypovolemia and inhibition of ROMK by WNK1 precludes K^+^ wasting. In contrast, K^+^ secretion via BK is independent on Na^+^ and is determined primarily by fluid delivery and activation of TRPV4 by flow shear stress [6,22]. The BK channel does not seem to substantially contribute to K^+^ secretion during hypovolemia due to reduced fluid delivery to the collecting duct. However, this mechanism is essential during high K^+^ intake associated with elevated flow to eliminate the excess of K^+^. At the same time, stimulation of ENaC by aldosterone would preclude urinary Na^+^ waste. Consistently, deletion of either BK or TRPV4 leads to hyperkalemia, when animals are subjected to high K^+^ diet [9,47]. It is quite possible that WNK1 sets up a balance between steady-state basal ROMK- and adaptive BK-dependent K^+^ secretion, therefore providing an additional level of flexibility to adjust K^+^ transport during various physiological states. Inappropriate WNK1 activation during Gordon’s syndrome compromises this balance leading not only to inhibition of ROMK, but also to decreased BK-dependent K^+^ secretion due to excessive NaCl (i.e., volume) retention by the distal convoluted tubule thus causing little flow in the collecting duct. Interestingly, thiazide diuretics are highly effective in reducing blood pressure and also by correcting hyperkalemia in PHAII patients [48]. It seems that NCC blockade leads to increased flow in the collecting duct to activate already augmented TRPV4 and BK thus promoting kaliuresis, which would not be possible otherwise due to WNK1-mediated inhibition of ROMK.

Our results (Figure 1, Figure 3, Figure 4, Figure 5, Figure 6 and Figure 7) demonstrate that inhibition of WNK1 with WNK-in-11 recapitulates the inhibitory effects of pan-selective WNK blocker, WNK463 with respect to TRPV4 function in both native and cultured collecting duct cells. This argues for the central role of WNK1 in determining TRPV4 expression in the kidney and its regulation by aldosterone, although WNK4 could also contribute as both isoforms synergize to control activity of other transporting systems in the ASDN, most notably NCC and ROMK [16]. Somewhat unexpectedly, the one previous study concluded that WNK4 inhibits TRPV4 activity by 30% upon over-expression in HEK293 cells [49]. The effect was indirect and was caused by a moderate reduction in TRPV4 membrane fraction, while the total expression was unchanged. WNK1 exhibited similar actions on TRPV4 and the effects of WNK1 and WNK4 were not additive. While the exact nature of this controversy is enigmatic, non-transfected HEK293 cells also exhibited marked responses to the used TRPV4 activators: 4α-PDD and hypotonicity (which substantially changes electrochemical driving force for ion fluxes) in the complete absence of TRPV4 expression [49]. Thus, it is possible that the observed inhibition of [Ca^2+^]_i_ responses could be attributed, at least to some extent, to the WNK actions on the non-identified targets also susceptible to stimulation by 4α-PDD and hypotonicity. In contrast, we show that deletion of TRPV4 completely abolishes [Ca^2+^]_i_ elevations in response to GSK1016790A (Figure S1), therefore excluding any off-target actions of the TRPV4 agonist. Furthermore, the previous study reports paradoxical and unexplained opposite effects of WNKs with kinase dead point mutations and kinase domain excisions on TRPV4 expression [49]. Our study demonstrates consistent inhibitory effects on TRPV4 activity and expression upon WNK inhibition/inactivation in native and cultured collecting ducts, as well as upon over-expression in CHO cells, which have no measurable background currents [34,38].

We show here that intact kinase activity is necessary for regulation of TRPV4 activity by WNK1 (Figure 8). Thus, kinase dead mutant K233M [41,42] not only precludes stimulation of TRPV4-mediated currents, but also reduces the current amplitude below the baseline, suggesting tonic regulation of the channel by endogenous WNK1. This is consistent with reduced GSK1016790A-mediated Ca^2+^ influx and decreased renal TRPV4 levels in mice treated with WNK463 (Figure 1). At this stage we cannot discriminate whether WNK1 is capable of directly phosphorylating TRPV4 or does this indirectly by targeting its downstream mediator. It was previously shown that the channel can be phosphorylated by protein kinases, including protein kinase A (PKA) and C (PKC), at several residues within the intracellular amino- and carboxyl- termini, leading to augmented TRPV4 activity and stimulation of trafficking to the plasma membrane [31,50]. Of interest, aldosterone-inducible serum glucocorticoid kinase 1 (SGK1) can also directly phosphorylate TRPV4 at Ser^824^ residue to increase channel activity in over-expression systems [51]. It is quite possible WNK1 and SGK1 belong to the same signaling pathway linking regulation of TRPV4 activity by aldosterone in the kidney. On the other hand, WNK1 does not directly phosphorylate NCC and BK, but exerts its stimulatory actions via the downstream effectors, namely SPAK and OSR1 [17]. Future studies are necessary to sort out the direct and indirect actions of WNK1 on TRPV4.

## 5. Conclusions

This study reveals a critical role of WNK1 in promoting TRPV4 functional expression and its stimulation by aldosterone in the kidney. Moreover, we have also examined the ramifications of systemic and local WNK1 blockade on TRPV4 with the recently developed orally active antagonists with high promise to become a new class of antihypertensive drugs in clinic. Apart from its prominent role in controlling Na^+^ balance, WNK signaling cascades also orchestrate a highly intricate interplay between renal sodium and potassium transport to maintain systemic homeostasis. TRPV4 appears to be a vital component of the flow-induced K^+^ secretion and is now an identified end-effector of the aldosterone-WNK1 signaling pathway in the ASDN.

## Data Availability

The data presented in this study are available in supplementary material.

## Supplementary Materials

The following are available online at https://www.mdpi.com/article/10.3390/cells10061482/s1, Figure S1: GSK1016790A fails to increase [Ca^2+^]_i_ in freshly isolated split-opened collecting ducts from TRPV4 -/- mice. Figure S2: Verification of specificity of anti-TRPV4 antibodies. Figure S3: Cortisone increases TRPV4-dependent Ca^2+^ influx in mpkCCD_c14_ cells. Figure S4: Verification of monitoring TRPV4-dependent current in CHO cells.
