# Supplementary material for: With-No-Lysine Kinase 1 (WNK1) Augments TRPV4 Function in the Aldosterone-Sensitive Distal Nephron
Source: Cells. 2021 Jun 12;10(6):1482. doi: 10.3390/cells10061482 (PMC8231605; doi:10.3390/cells10061482)
Supplement: Supplementary file 1 [file cells-10-01482-s001.zip › cells-1218256-supplementary.pdf]

SUPPLEMENTARY FIGURES

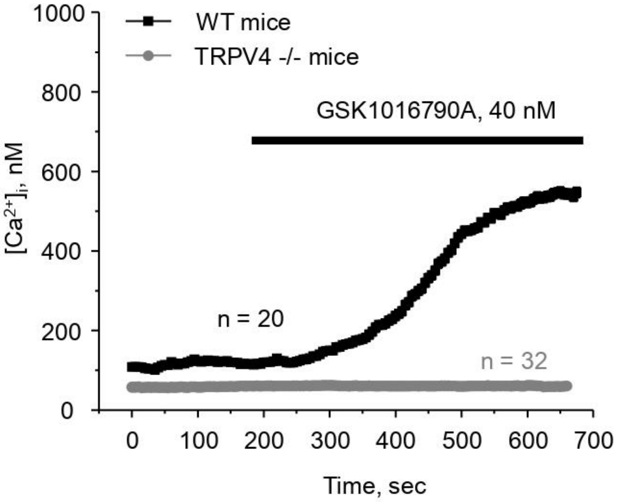

Supplementary Figure S1. GSK1016790A fails to increase $[Ca^{2+}]_i$ in freshly isolated split-opened collecting ducts from TRPV4 -/- mice. The averaged time-courses of $[Ca^{2+}]_i$ changes upon application of 40 nM GSK1016790A (shown with the bar on top) in individual cells within split-opened area of freshly isolated collecting ducts from WT (black) and TRPV4-/- (gray) mice. Number of individual experiments is shown.

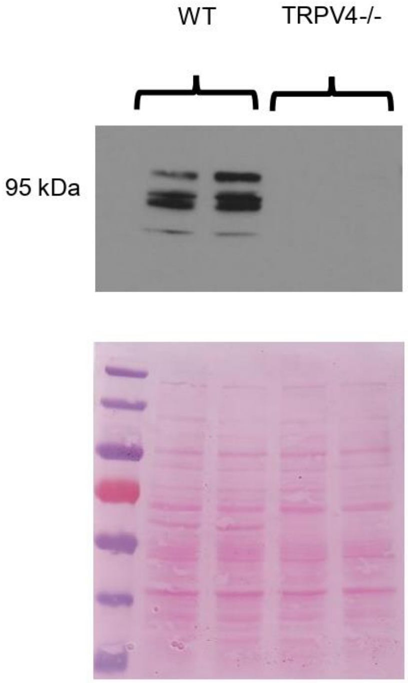

Supplementary Figure S2. Verification of specificity of anti-TRPV4 antibodies. Representative Western blot from whole kidney lysates of WT and TRPV4 -/- mice probed with anti-TRPV4 antibodies. The main TRPV4 reporting signal appears as a duplet of glycosylated and non-glycosylated forms around 95 kDa. The Ponceau red staining of the same nitrocellulose membrane demonstrating equal protein loading is shown on the bottom panel.

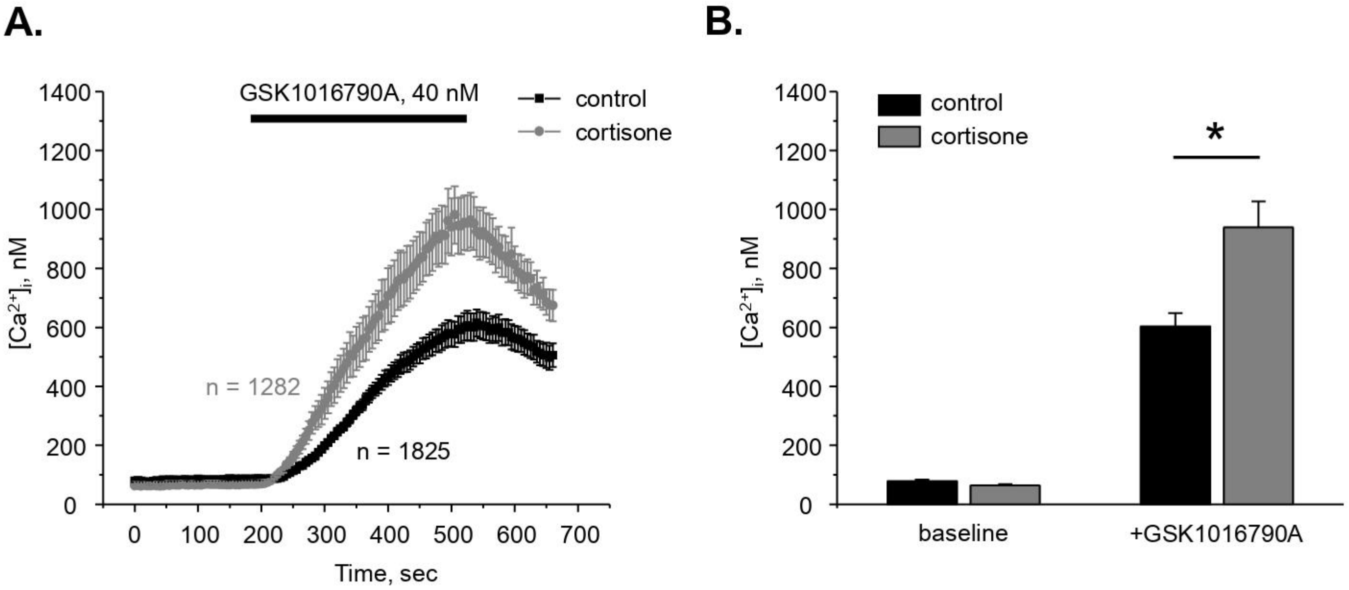

Supplementary Figure S3. Cortisone increases TRPV4-dependent Ca^{2+} influx in mpkCCD$_{c14}$ cells. (**A**) The averaged time-courses of $[Ca^{2+}]_i$ changes upon application of 40 nM GSK1016790A (shown with the bar on top) in individual mpkCCD$_{c14}$ cells maintained on standard (control) conditions and after pre-incubation with cortisone. (**B**) Summary graph comparing $[Ca^{2+}]_i$ values in individual mpkCCD$_{c14}$ cells in the control and following cortisone treatment at the baseline and after GSK1016790A application. *-significant difference ($p < 0.05$) between groups is indicated with a line.

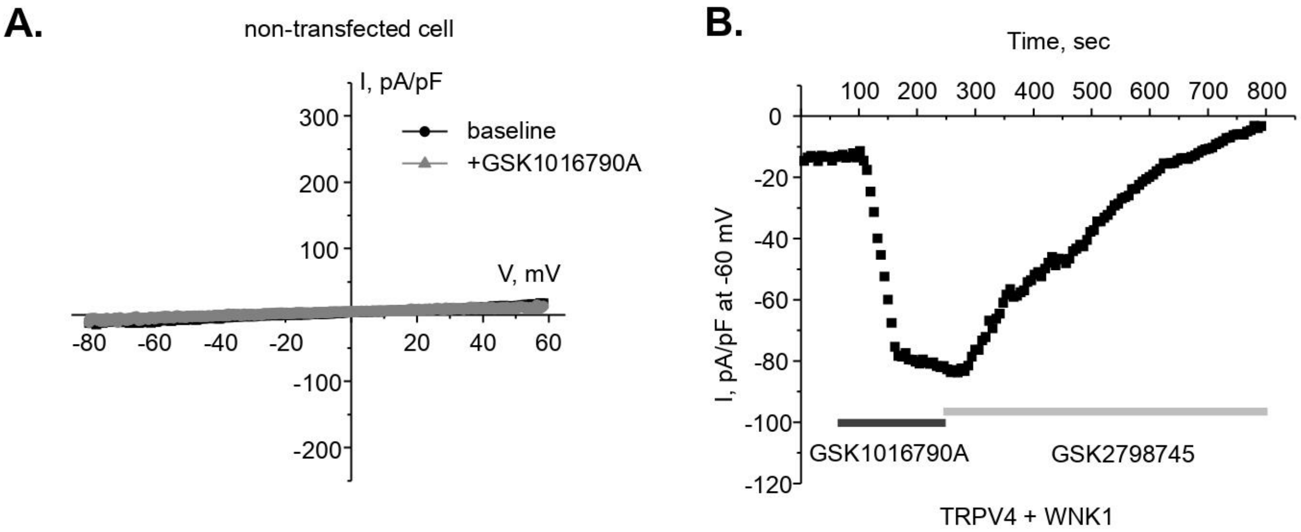

Supplementary Figure S4. Verification of monitoring TRPV4-dependent current in CHO cells. (**A**) Representative macroscopic whole-cell current-voltage (I-V) relations in non-transfected Chinese Hamster Ovary (CHO) cells at the baseline (black) and following application of TRPV4 agonist GSK1016790A (40 nM) for 5 min (gray). Currents were evoked by a voltage ramp from −80 to +60 mV for 1 sec. (**B**) Representative time course of changes in macroscopic whole-cell current amplitude at −60 mV from a CHO cell transfected with TRPV4 and WNK1 upon serial application of TRPV4 agonist, GSK1016790A (40 nM) and TRPV4 antagonist GSK2798745 (40 nM). The application times are shown with gray and light gray bars, respectively.
